# The mixed-valent copper thiol­ate complex hexa­kis­{μ_3_-2-[(1,3-dimethyl­imidazol­idene)amino]­benzene­thiol­ato}dicopper(II)tetra­copper(I) bis­(hexa­fluoridophosphate) acetonitrile disolvate dichloro­methane disolvate

**DOI:** 10.1107/S1600536812050428

**Published:** 2012-12-15

**Authors:** Adam Neuba, Ulrich Flörke, Gerald Henkel

**Affiliations:** a Universität Paderborn, Fakultät für Naturwissenschaften, Department Chemie, Warburger Strasse 100, 33098 Paderborn

## Abstract

The mol­ecular structure of the title compound, [Cu_4_^I^Cu_2_^II^(C_11_H_14_N_3_S)_6_](PF_6_)_2_·2CH_3_CN·2CH_2_Cl_2_, shows a mixed-valent copper(I/II) thiol­ate complex with a distorted tetra­hedral coordination of the Cu^I^ and Cu^II^ cations by one guanidine N atom and three S atoms each. Characteristic features of the Cu_6_S_6_ skeleton are a total of six chemically identical μ_3_-thiol­ate bridges and almost planar Cu_2_S_2_ units with a maximum deviation of 0.110 (1) Å from the best plane. Each Cu_2_S_2_ unit then shares common Cu–S edges with a neighbouring unit; the enclosed dihedral angle is 60.14 (2)°. The geometric centre of the Cu_6_S_6_ cation lies on a crystallographic inversion centre. Cu—S bond lengths range from 2.294 (1) to 2.457 (1) Å, Cu—N bond lengths from 2.005 (3) to 2.018 (3) Å and the non-bonding Cu⋯Cu distances from 2.5743 (7) to 2.5892 (6) Å. C—H⋯F hydrogen-bond inter­actions occur between the PF_6_^−^ anion and the complex mol­ecule and between the PF_6_^−^ anion and the acetonitrile solvent mol­ecule.

## Related literature

For bifunctional peralkyl­ated guanidine ligands, see: Bienemann *et al.* (2011 ▶); Börner *et al.* (2009 ▶); Herres-Pawlis *et al.* (2005 ▶, 2009 ▶); Neuba *et al.* (2008 ▶, 2010 ▶); Pohl *et al.* (2000 ▶); Raab *et al.* (2003 ▶); Wittmann *et al.* (2001 ▶). This investigation is part of our work towards bi- and polyfunctional guanidine–sulfur hybrids to mimic the structural and physical, as well as functional characteristics of the Cu_A_ center in cytochrom c oxidase and N_2_O reductase, see: Neuba *et al.* (2011 ▶, 2012 ▶).
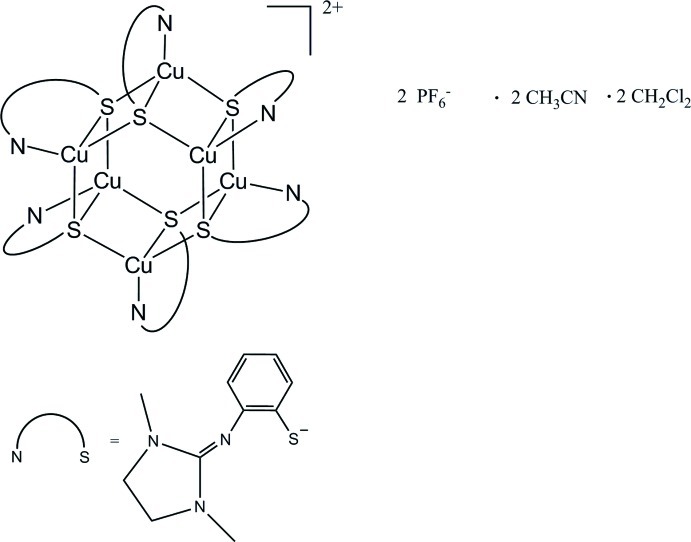


## Experimental

### 

#### Crystal data

[Cu_6_(C_11_H_14_N_3_S)_6_](PF_6_)_2_·2C_2_H_3_N·2CH_2_Cl_2_
*M* *_r_* = 2245.01
Triclinic, 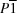

*a* = 13.019 (2) Å
*b* = 13.687 (3) Å
*c* = 13.875 (3) Å
α = 108.371 (4)°
β = 94.768 (4)°
γ = 102.691 (4)°
*V* = 2257.8 (7) Å^3^
*Z* = 1
Mo *K*α radiation
μ = 1.76 mm^−1^
*T* = 120 K
0.44 × 0.38 × 0.25 mm

#### Data collection

Bruker SMART APEX diffractometer
Absorption correction: multi-scan (*SADABS*; Sheldrick, 2004 ▶) *T*_min_ = 0.512, *T*_max_ = 0.668
18315 measured reflections
10640 independent reflections
8260 reflections with *I* > 2σ(*I*)
*R*_int_ = 0.031

#### Refinement

*R*[*F* ^2^ > 2σ(*F* ^2^)] = 0.048
*wR*(*F* ^2^) = 0.138
*S* = 1.03
10640 reflections
557 parameters
H-atom parameters constrained
Δρ_max_ = 0.97 e Å^−3^
Δρ_min_ = −0.90 e Å^−3^

### 

Data collection: *SMART* (Bruker, 2002 ▶); cell refinement: *SAINT* (Bruker, 2002 ▶); data reduction: *SAINT*; program(s) used to solve structure: *SHELXS97* (Sheldrick, 2008 ▶); program(s) used to refine structure: *SHELXL97* (Sheldrick, 2008 ▶); molecular graphics: *SHELXTL* (Sheldrick, 2008 ▶); software used to prepare material for publication: *SHELXTL*.

## Supplementary Material

Crystal structure: contains datablock(s) I, global. DOI: 10.1107/S1600536812050428/nr2037sup1.cif

Structure factors: contains datablock(s) I. DOI: 10.1107/S1600536812050428/nr2037Isup2.hkl

Additional supplementary materials:  crystallographic information; 3D view; checkCIF report

## Figures and Tables

**Table 1:** Hydrogen-bond geometry (Å, °)

*D*—H⋯*A*	*D*—H	H⋯*A*	*D*⋯*A*	*D*—H⋯*A*
C31—H31*A*⋯F6^i^	0.95	2.49	3.290 (5)	141
C102—H10*F*⋯F4^ii^	0.98	2.43	3.173 (6)	133
C102—H10*E*⋯F1^iii^	0.98	2.44	3.187 (7)	133

Symmetry codes: (i) 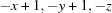; (ii) 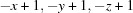; (iii) 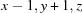.

**Fig. 1.**
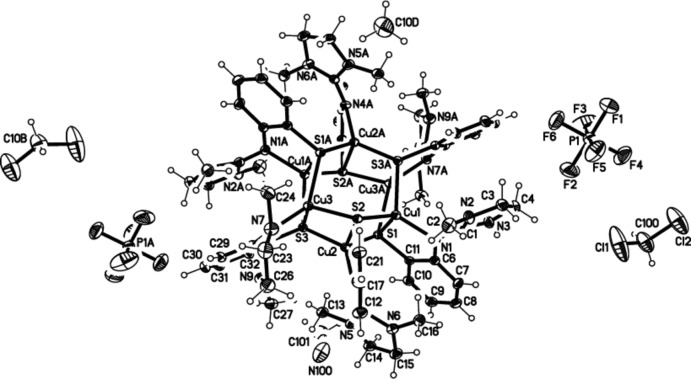
Molecular structure of the title compound. Anistropic displacement parameters are shown at the 50% probability level.

**Fig. 2.**
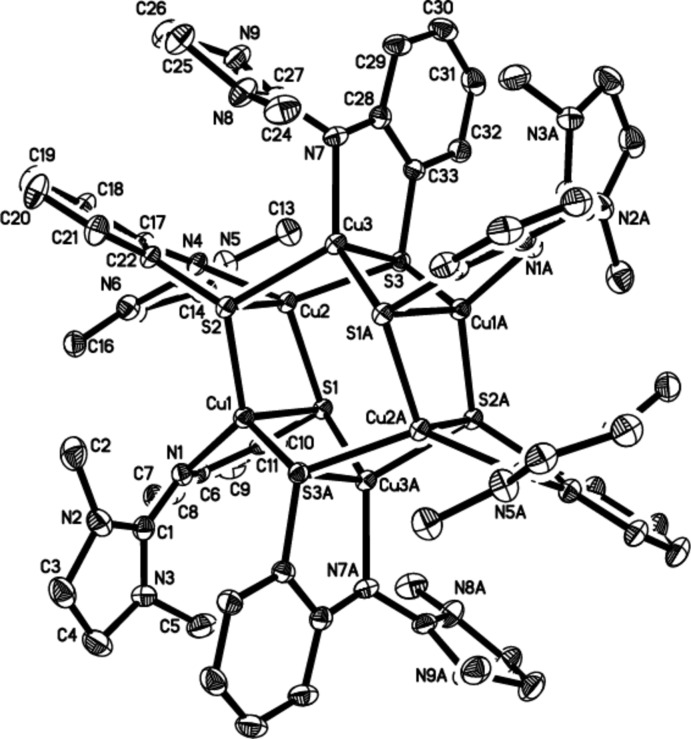
Structure of the cation, H-atoms omitted.

## supplementary crystallographic information

### Experimental

The reaction of *N*-(1,3-dimethylimidazolidin-2-ylidene)-2-(tritylthio)aniline (510 mg, 1.1 mmol) with [Cu(MeCN)_4_](PF_6_) (186.2 mg, 0.5 mmol) dissolved in 5 ml of *ABS*. MeCN leads to a deep blue/green solution which was stirred for 30 min. at room temperature followed by heating under reflux for 30 min. After cooling the solution was filtered. Diffusion of Et_2_O into the filtrate afforded a black precipitate which was dissolved in MeCN/CH_2_Cl_2_ (5:1). Slow diffusion of Et_2_O in this solution afforded black crystals of [Cu_6_(C_11_H_14_N_3_S)_6_](PF_6_)_2_ 2 CH_2_Cl_2_ 2 MeCN suitable for X-ray diffraction. The mechanism of the complex formation is not completely understood (Neuba *et al.*, 2011). We suppose a combination of homo- and heterolytic cleavage of the S-CPh_3_ bond with partial oxidation of copper(I) to copper(II) during the reaction.

### Refinement

Hydrogen atoms were clearly identified in difference syntheses, refined at idealized positions riding on the carbon atoms with isotropic displacement parameters *U*_iso_(H) = 1.2U(C_eq_) or 1.5U(–CH_3_) and C–H 0.95–0.99 Å. All CH_3_ hydrogen atoms were allowed to rotate but not to tip.

### Crystal data

**Table:**

[Cu_6_(C_11_H_14_N_3_S)_6_](PF_6_)_2_·2C_2_H_3_N·2CH_2_Cl_2_	*Z* = 1
*M**_r_* = 2245.01	*F*(000) = 1142
Triclinic, *P*1	*D*_x_ = 1.651 Mg m^−^^3^
Hall symbol: -P 1	Mo *K*α radiation, λ = 0.71073 Å
*a* = 13.019 (2) Å	Cell parameters from 5162 reflections
*b* = 13.687 (3) Å	θ = 2.4–27.4°
*c* = 13.875 (3) Å	µ = 1.76 mm^−^^1^
α = 108.371 (4)°	*T* = 120 K
β = 94.768 (4)°	Prism, black
γ = 102.691 (4)°	0.44 × 0.38 × 0.25 mm
*V* = 2257.8 (7) Å^3^	

### Data collection

**Table:**

Bruker SMART APEX diffractometer	10640 independent reflections
Radiation source: sealed tube	8260 reflections with *I* > 2σ(*I*)
Graphite monochromator	*R*_int_ = 0.031
φ and ω scans	θ_max_ = 27.9°, θ_min_ = 1.6°
Absorption correction: multi-scan (*SADABS*; Sheldrick, 2004)	*h* = −13→17
*T*_min_ = 0.512, *T*_max_ = 0.668	*k* = −17→18
18315 measured reflections	*l* = −18→18

### Refinement

**Table:**

Refinement on *F*^2^	Primary atom site location: structure-invariant direct methods
Least-squares matrix: full	Secondary atom site location: difference Fourier map
*R*[*F*^2^ > 2σ(*F*^2^)] = 0.048	Hydrogen site location: difference Fourier map
*wR*(*F*^2^) = 0.138	H-atom parameters constrained
*S* = 1.03	*w* = 1/[σ^2^(*F*_o_^2^) + (0.0733*P*)^2^ + 1.372*P*] where *P* = (*F*_o_^2^ + 2*F*_c_^2^)/3
10640 reflections	(Δ/σ)_max_ = 0.007
557 parameters	Δρ_max_ = 0.97 e Å^−^^3^
0 restraints	Δρ_min_ = −0.90 e Å^−^^3^

### Special details

**Table:**

Geometry. All e.s.d.'s (except the e.s.d. in the dihedral angle between two l.s. planes) are estimated using the full covariance matrix. The cell e.s.d.'s are taken into account individually in the estimation of e.s.d.'s in distances, angles and torsion angles; correlations between e.s.d.'s in cell parameters are only used when they are defined by crystal symmetry. An approximate (isotropic) treatment of cell e.s.d.'s is used for estimating e.s.d.'s involving l.s. planes.
Refinement. Refinement of *F*^2^ against ALL reflections. The weighted *R*-factor *wR* and goodness of fit *S* are based on *F*^2^, conventional *R*-factors *R* are based on *F*, with *F* set to zero for negative *F*^2^. The threshold expression of *F*^2^ > σ(*F*^2^) is used only for calculating *R*-factors(gt) *etc*. and is not relevant to the choice of reflections for refinement. *R*-factors based on *F*^2^ are statistically about twice as large as those based on *F*, and *R*- factors based on ALL data will be even larger.

### Fractional atomic coordinates and isotropic or equivalent isotropic displacement parameters (Å^2^)

**Table:**

	*x*	*y*	*z*	*U*_iso_*/*U*_eq_	
Cu1	0.62734 (3)	0.54975 (3)	0.13981 (3)	0.01963 (11)	
Cu2	0.54012 (3)	0.67921 (3)	0.08413 (3)	0.01902 (10)	
Cu3	0.55066 (3)	0.59234 (3)	−0.10848 (3)	0.01968 (11)	
S1	0.45590 (6)	0.58501 (6)	0.18015 (6)	0.01848 (16)	
S2	0.69051 (6)	0.61789 (6)	0.01818 (6)	0.01843 (16)	
S3	0.38980 (6)	0.62400 (6)	−0.03894 (6)	0.01842 (16)	
N1	0.6730 (2)	0.5961 (2)	0.2925 (2)	0.0223 (6)	
N2	0.8297 (3)	0.5366 (3)	0.2911 (3)	0.0335 (7)	
N3	0.7236 (3)	0.4993 (3)	0.3983 (2)	0.0304 (7)	
N4	0.6463 (2)	0.8206 (2)	0.1608 (2)	0.0204 (6)	
N5	0.5361 (3)	0.9110 (3)	0.2603 (2)	0.0289 (7)	
N6	0.6917 (3)	0.9194 (2)	0.3424 (2)	0.0279 (7)	
N7	0.5452 (2)	0.7096 (2)	−0.1660 (2)	0.0230 (6)	
N8	0.7187 (3)	0.7395 (3)	−0.2063 (2)	0.0323 (7)	
N9	0.6661 (3)	0.8836 (3)	−0.1248 (2)	0.0322 (7)	
C1	0.7392 (3)	0.5479 (3)	0.3274 (3)	0.0249 (7)	
C2	0.8823 (3)	0.5962 (4)	0.2311 (3)	0.0387 (9)	
H2A	0.8430	0.6476	0.2239	0.058*	
H2B	0.8839	0.5470	0.1628	0.058*	
H2C	0.9554	0.6342	0.2661	0.058*	
C3	0.8860 (3)	0.4806 (3)	0.3424 (3)	0.0378 (9)	
H3A	0.9553	0.5278	0.3831	0.045*	
H3B	0.8985	0.4166	0.2919	0.045*	
C4	0.8101 (4)	0.4500 (4)	0.4119 (3)	0.0401 (10)	
H4A	0.7829	0.3716	0.3906	0.048*	
H4B	0.8458	0.4782	0.4846	0.048*	
C5	0.6194 (4)	0.4573 (3)	0.4214 (3)	0.0378 (9)	
H5A	0.5634	0.4623	0.3728	0.057*	
H5B	0.6138	0.4988	0.4917	0.057*	
H5C	0.6108	0.3825	0.4153	0.057*	
C6	0.6089 (3)	0.6453 (3)	0.3558 (2)	0.0222 (7)	
C7	0.6436 (3)	0.7019 (3)	0.4631 (3)	0.0290 (8)	
H7A	0.7128	0.7049	0.4935	0.035*	
C8	0.5792 (3)	0.7521 (3)	0.5236 (3)	0.0325 (9)	
H8A	0.6045	0.7891	0.5950	0.039*	
C9	0.4774 (3)	0.7495 (3)	0.4817 (3)	0.0312 (8)	
H9A	0.4330	0.7836	0.5243	0.037*	
C10	0.4412 (3)	0.6965 (3)	0.3767 (3)	0.0252 (7)	
H10A	0.3717	0.6941	0.3474	0.030*	
C11	0.5070 (3)	0.6467 (3)	0.3142 (2)	0.0207 (7)	
C12	0.6285 (3)	0.8814 (3)	0.2505 (3)	0.0219 (7)	
C13	0.4489 (3)	0.8864 (3)	0.1790 (3)	0.0311 (8)	
H13A	0.4761	0.8797	0.1141	0.047*	
H13B	0.4123	0.9435	0.1947	0.047*	
H13C	0.3986	0.8190	0.1726	0.047*	
C14	0.5292 (3)	0.9639 (3)	0.3666 (3)	0.0304 (8)	
H14A	0.4766	0.9177	0.3918	0.036*	
H14B	0.5092	1.0316	0.3759	0.036*	
C15	0.6431 (3)	0.9841 (3)	0.4219 (3)	0.0323 (8)	
H15A	0.6815	1.0605	0.4446	0.039*	
H15B	0.6424	0.9604	0.4823	0.039*	
C16	0.7869 (3)	0.8878 (3)	0.3696 (3)	0.0306 (8)	
H16A	0.7860	0.8187	0.3191	0.046*	
H16B	0.7880	0.8819	0.4382	0.046*	
H16C	0.8507	0.9414	0.3696	0.046*	
C17	0.7492 (3)	0.8350 (3)	0.1357 (3)	0.0222 (7)	
C18	0.8246 (3)	0.9346 (3)	0.1699 (3)	0.0261 (7)	
H18A	0.8076	0.9944	0.2162	0.031*	
C19	0.9228 (3)	0.9478 (3)	0.1380 (3)	0.0340 (9)	
H19A	0.9724	1.0161	0.1627	0.041*	
C20	0.9495 (3)	0.8614 (3)	0.0697 (3)	0.0379 (9)	
H20A	1.0171	0.8704	0.0478	0.045*	
C21	0.8772 (3)	0.7629 (3)	0.0345 (3)	0.0281 (8)	
H21A	0.8950	0.7042	−0.0126	0.034*	
C22	0.7778 (3)	0.7479 (3)	0.0668 (3)	0.0215 (7)	
C23	0.6375 (3)	0.7756 (3)	−0.1655 (3)	0.0256 (7)	
C24	0.7053 (4)	0.6345 (3)	−0.2809 (3)	0.0380 (9)	
H24A	0.6311	0.5930	−0.2913	0.057*	
H24B	0.7528	0.5983	−0.2555	0.057*	
H24C	0.7228	0.6409	−0.3463	0.057*	
C25	0.8059 (4)	0.8277 (4)	−0.2029 (4)	0.0434 (11)	
H25A	0.8059	0.8367	−0.2709	0.052*	
H25B	0.8757	0.8173	−0.1803	0.052*	
C26	0.7820 (4)	0.9227 (3)	−0.1238 (3)	0.0413 (10)	
H26A	0.8238	0.9399	−0.0549	0.050*	
H26B	0.7974	0.9865	−0.1448	0.050*	
C27	0.6231 (4)	0.9424 (3)	−0.0361 (3)	0.0352 (9)	
H27A	0.5483	0.9383	−0.0578	0.053*	
H27B	0.6645	1.0171	−0.0094	0.053*	
H27C	0.6280	0.9108	0.0179	0.053*	
C28	0.4523 (3)	0.7436 (3)	−0.1644 (2)	0.0229 (7)	
C29	0.4281 (3)	0.8071 (3)	−0.2221 (3)	0.0296 (8)	
H29A	0.4796	0.8321	−0.2594	0.035*	
C30	0.3324 (4)	0.8333 (3)	−0.2255 (3)	0.0348 (9)	
H30A	0.3188	0.8760	−0.2649	0.042*	
C31	0.2559 (3)	0.7986 (3)	−0.1726 (3)	0.0323 (8)	
H31A	0.1900	0.8175	−0.1753	0.039*	
C32	0.2754 (3)	0.7358 (3)	−0.1152 (3)	0.0267 (7)	
H32A	0.2226	0.7119	−0.0785	0.032*	
C33	0.3722 (3)	0.7076 (3)	−0.1111 (2)	0.0211 (7)	
P1	0.99842 (9)	0.22393 (9)	0.42113 (8)	0.0350 (2)	
F1	1.0487 (4)	0.1249 (3)	0.3935 (3)	0.0978 (14)	
F2	0.9544 (3)	0.3243 (3)	0.4447 (3)	0.0989 (14)	
F3	0.8879 (3)	0.1500 (4)	0.4215 (3)	0.1167 (18)	
F4	1.0369 (3)	0.2506 (3)	0.5397 (2)	0.0782 (11)	
F5	1.1110 (3)	0.2967 (3)	0.4188 (2)	0.0779 (10)	
F6	0.9664 (3)	0.1973 (3)	0.3009 (2)	0.0729 (10)	
C100	0.8296 (5)	0.4191 (6)	0.8897 (5)	0.0758 (19)	
H10B	0.7527	0.3814	0.8732	0.091*	
H10C	0.8400	0.4814	0.9533	0.091*	
Cl1	0.86150 (19)	0.4642 (3)	0.7934 (2)	0.1522 (14)	
Cl2	0.89884 (19)	0.3361 (2)	0.91445 (19)	0.1187 (9)	
N100	0.2667 (4)	0.9850 (5)	0.3182 (4)	0.0770 (16)	
C101	0.1838 (5)	0.9396 (5)	0.3266 (4)	0.0587 (14)	
C102	0.0791 (5)	0.8897 (5)	0.3378 (5)	0.0701 (16)	
H10D	0.0340	0.8557	0.2699	0.105*	
H10E	0.0484	0.9434	0.3817	0.105*	
H10F	0.0833	0.8356	0.3694	0.105*	

### Atomic displacement parameters (Å^2^)

**Table:**

	*U*^11^	*U*^22^	*U*^33^	*U*^12^	*U*^13^	*U*^23^
Cu1	0.0198 (2)	0.0180 (2)	0.0206 (2)	0.00440 (16)	0.00325 (15)	0.00639 (16)
Cu2	0.0183 (2)	0.0166 (2)	0.0207 (2)	0.00298 (15)	0.00323 (15)	0.00534 (15)
Cu3	0.0200 (2)	0.0167 (2)	0.0216 (2)	0.00346 (16)	0.00395 (16)	0.00638 (16)
S1	0.0183 (4)	0.0164 (4)	0.0198 (4)	0.0035 (3)	0.0037 (3)	0.0055 (3)
S2	0.0171 (4)	0.0158 (4)	0.0209 (4)	0.0027 (3)	0.0036 (3)	0.0052 (3)
S3	0.0174 (4)	0.0168 (4)	0.0208 (4)	0.0043 (3)	0.0025 (3)	0.0064 (3)
N1	0.0211 (15)	0.0234 (15)	0.0213 (13)	0.0043 (12)	0.0016 (11)	0.0074 (11)
N2	0.0283 (17)	0.0384 (19)	0.0370 (17)	0.0124 (15)	0.0019 (14)	0.0157 (15)
N3	0.0328 (18)	0.0264 (17)	0.0333 (16)	0.0051 (13)	0.0003 (13)	0.0152 (14)
N4	0.0204 (14)	0.0142 (13)	0.0230 (13)	0.0028 (11)	0.0048 (11)	0.0024 (11)
N5	0.0289 (17)	0.0315 (17)	0.0243 (15)	0.0134 (14)	0.0056 (12)	0.0028 (13)
N6	0.0297 (17)	0.0254 (16)	0.0245 (15)	0.0049 (13)	0.0047 (12)	0.0042 (12)
N7	0.0272 (16)	0.0191 (14)	0.0251 (14)	0.0050 (12)	0.0061 (12)	0.0109 (12)
N8	0.0285 (17)	0.0316 (18)	0.0360 (17)	0.0027 (14)	0.0144 (14)	0.0117 (14)
N9	0.0356 (19)	0.0243 (17)	0.0313 (16)	−0.0031 (13)	0.0074 (14)	0.0092 (13)
C1	0.0249 (18)	0.0219 (18)	0.0233 (16)	0.0026 (14)	0.0008 (13)	0.0049 (14)
C2	0.025 (2)	0.050 (3)	0.041 (2)	0.0100 (18)	0.0087 (17)	0.0144 (19)
C3	0.036 (2)	0.031 (2)	0.042 (2)	0.0138 (18)	−0.0060 (18)	0.0056 (17)
C4	0.047 (3)	0.035 (2)	0.038 (2)	0.0153 (19)	−0.0057 (19)	0.0134 (18)
C5	0.049 (3)	0.031 (2)	0.033 (2)	0.0030 (18)	0.0069 (18)	0.0152 (17)
C6	0.0264 (18)	0.0170 (16)	0.0216 (16)	0.0024 (13)	0.0037 (13)	0.0069 (13)
C7	0.031 (2)	0.028 (2)	0.0222 (17)	0.0002 (15)	−0.0023 (14)	0.0069 (14)
C8	0.047 (2)	0.0237 (19)	0.0208 (17)	0.0040 (17)	0.0044 (16)	0.0040 (14)
C9	0.047 (2)	0.0236 (19)	0.0260 (18)	0.0131 (17)	0.0149 (16)	0.0073 (15)
C10	0.0297 (19)	0.0213 (18)	0.0261 (17)	0.0066 (14)	0.0094 (14)	0.0089 (14)
C11	0.0269 (18)	0.0144 (16)	0.0198 (15)	0.0032 (13)	0.0049 (13)	0.0058 (12)
C12	0.0246 (18)	0.0135 (15)	0.0252 (16)	0.0000 (13)	0.0025 (13)	0.0072 (13)
C13	0.032 (2)	0.030 (2)	0.0303 (19)	0.0153 (16)	0.0052 (15)	0.0044 (15)
C14	0.037 (2)	0.0248 (19)	0.0276 (18)	0.0094 (16)	0.0100 (16)	0.0050 (15)
C15	0.041 (2)	0.030 (2)	0.0233 (17)	0.0091 (17)	0.0093 (16)	0.0037 (15)
C16	0.0269 (19)	0.030 (2)	0.0310 (19)	0.0049 (15)	0.0002 (15)	0.0083 (16)
C17	0.0228 (17)	0.0194 (17)	0.0220 (16)	0.0030 (13)	0.0038 (13)	0.0054 (13)
C18	0.0256 (19)	0.0216 (18)	0.0267 (17)	−0.0004 (14)	0.0059 (14)	0.0063 (14)
C19	0.031 (2)	0.024 (2)	0.038 (2)	−0.0053 (15)	0.0039 (16)	0.0067 (16)
C20	0.0208 (19)	0.038 (2)	0.048 (2)	−0.0006 (16)	0.0128 (17)	0.0100 (19)
C21	0.0213 (18)	0.029 (2)	0.0316 (18)	0.0064 (15)	0.0095 (14)	0.0052 (15)
C22	0.0196 (16)	0.0173 (16)	0.0243 (16)	−0.0014 (13)	0.0016 (13)	0.0074 (13)
C23	0.0306 (19)	0.0223 (18)	0.0257 (17)	0.0032 (14)	0.0060 (14)	0.0127 (14)
C24	0.045 (3)	0.035 (2)	0.041 (2)	0.0154 (19)	0.0193 (19)	0.0165 (18)
C25	0.031 (2)	0.048 (3)	0.051 (3)	−0.0031 (19)	0.0103 (19)	0.025 (2)
C26	0.040 (2)	0.035 (2)	0.041 (2)	−0.0085 (18)	0.0041 (18)	0.0153 (19)
C27	0.045 (2)	0.024 (2)	0.0312 (19)	0.0059 (17)	0.0042 (17)	0.0050 (16)
C28	0.0277 (18)	0.0163 (16)	0.0201 (15)	0.0034 (13)	0.0012 (13)	0.0023 (13)
C29	0.044 (2)	0.0202 (18)	0.0263 (17)	0.0090 (16)	0.0053 (16)	0.0098 (14)
C30	0.050 (3)	0.025 (2)	0.0313 (19)	0.0127 (18)	−0.0006 (17)	0.0121 (16)
C31	0.030 (2)	0.029 (2)	0.038 (2)	0.0140 (16)	−0.0021 (16)	0.0087 (16)
C32	0.0253 (19)	0.0238 (18)	0.0319 (18)	0.0082 (15)	0.0015 (15)	0.0101 (15)
C33	0.0260 (18)	0.0173 (16)	0.0185 (15)	0.0052 (13)	0.0010 (13)	0.0049 (12)
P1	0.0344 (6)	0.0298 (6)	0.0353 (5)	0.0065 (4)	−0.0010 (4)	0.0064 (4)
F1	0.133 (4)	0.066 (2)	0.091 (3)	0.060 (3)	−0.006 (2)	0.005 (2)
F2	0.096 (3)	0.070 (3)	0.112 (3)	0.056 (2)	−0.019 (2)	−0.008 (2)
F3	0.084 (3)	0.099 (3)	0.110 (3)	−0.043 (2)	0.042 (2)	−0.004 (2)
F4	0.111 (3)	0.077 (2)	0.0349 (15)	0.005 (2)	0.0047 (16)	0.0191 (15)
F5	0.053 (2)	0.093 (3)	0.063 (2)	−0.0158 (18)	0.0042 (16)	0.0190 (18)
F6	0.070 (2)	0.088 (3)	0.0459 (16)	0.0163 (19)	−0.0128 (15)	0.0108 (16)
C100	0.065 (4)	0.095 (5)	0.104 (5)	0.049 (4)	0.040 (4)	0.058 (4)
Cl1	0.1117 (17)	0.280 (4)	0.207 (3)	0.136 (2)	0.1072 (19)	0.200 (3)
Cl2	0.1235 (17)	0.164 (2)	0.165 (2)	0.1046 (17)	0.0989 (16)	0.1237 (19)
N100	0.054 (3)	0.116 (5)	0.056 (3)	0.021 (3)	0.011 (2)	0.023 (3)
C101	0.052 (3)	0.090 (4)	0.038 (3)	0.022 (3)	0.004 (2)	0.026 (3)
C102	0.073 (4)	0.071 (4)	0.079 (4)	0.015 (3)	0.030 (3)	0.040 (3)

### Geometric parameters (Å, º)

**Table:**

Cu1—N1	2.005 (3)	C10—C11	1.398 (5)
Cu1—S2	2.2977 (9)	C10—H10A	0.9500
Cu1—S3^i^	2.2981 (10)	C13—H13A	0.9800
Cu1—S1	2.4573 (10)	C13—H13B	0.9800
Cu2—N4	2.018 (3)	C13—H13C	0.9800
Cu2—S3	2.3017 (9)	C14—C15	1.536 (6)
Cu2—S1	2.3117 (9)	C14—H14A	0.9900
Cu2—S2	2.4312 (9)	C14—H14B	0.9900
Cu3—N7	2.017 (3)	C15—H15A	0.9900
Cu3—S1^i^	2.2939 (10)	C15—H15B	0.9900
Cu3—S2	2.3079 (9)	C16—H16A	0.9800
Cu3—S3	2.4446 (10)	C16—H16B	0.9800
S1—C11	1.782 (3)	C16—H16C	0.9800
S1—Cu3^i^	2.2939 (10)	C17—C18	1.405 (5)
S2—C22	1.772 (3)	C17—C22	1.418 (5)
S3—C33	1.779 (3)	C18—C19	1.381 (5)
S3—Cu1^i^	2.2981 (10)	C18—H18A	0.9500
N1—C1	1.339 (4)	C19—C20	1.396 (6)
N1—C6	1.385 (4)	C19—H19A	0.9500
N2—C1	1.340 (5)	C20—C21	1.376 (5)
N2—C2	1.450 (5)	C20—H20A	0.9500
N2—C3	1.461 (5)	C21—C22	1.398 (5)
N3—C1	1.356 (4)	C21—H21A	0.9500
N3—C5	1.456 (5)	C24—H24A	0.9800
N3—C4	1.462 (5)	C24—H24B	0.9800
N4—C12	1.333 (4)	C24—H24C	0.9800
N4—C17	1.400 (4)	C25—C26	1.525 (7)
N5—C12	1.355 (5)	C25—H25A	0.9900
N5—C13	1.433 (5)	C25—H25B	0.9900
N5—C14	1.443 (4)	C26—H26A	0.9900
N6—C12	1.339 (4)	C26—H26B	0.9900
N6—C16	1.458 (5)	C27—H27A	0.9800
N6—C15	1.465 (5)	C27—H27B	0.9800
N7—C23	1.333 (4)	C27—H27C	0.9800
N7—C28	1.387 (5)	C28—C33	1.417 (5)
N8—C23	1.354 (5)	C28—C29	1.418 (5)
N8—C24	1.445 (5)	C29—C30	1.371 (6)
N8—C25	1.450 (5)	C29—H29A	0.9500
N9—C23	1.357 (5)	C30—C31	1.374 (6)
N9—C27	1.470 (5)	C30—H30A	0.9500
N9—C26	1.483 (5)	C31—C32	1.390 (5)
C2—H2A	0.9800	C31—H31A	0.9500
C2—H2B	0.9800	C32—C33	1.399 (5)
C2—H2C	0.9800	C32—H32A	0.9500
C3—C4	1.522 (6)	P1—F2	1.555 (3)
C3—H3A	0.9900	P1—F3	1.568 (4)
C3—H3B	0.9900	P1—F4	1.579 (3)
C4—H4A	0.9900	P1—F1	1.587 (3)
C4—H4B	0.9900	P1—F6	1.589 (3)
C5—H5A	0.9800	P1—F5	1.590 (3)
C5—H5B	0.9800	C100—Cl1	1.685 (6)
C5—H5C	0.9800	C100—Cl2	1.692 (6)
C6—C11	1.408 (5)	C100—H10B	0.9900
C6—C7	1.424 (5)	C100—H10C	0.9900
C7—C8	1.373 (5)	N100—C101	1.156 (7)
C7—H7A	0.9500	C101—C102	1.428 (8)
C8—C9	1.390 (6)	C102—H10D	0.9800
C8—H8A	0.9500	C102—H10E	0.9800
C9—C10	1.393 (5)	C102—H10F	0.9800
C9—H9A	0.9500		
			
N1—Cu1—S2	131.91 (9)	H13A—C13—H13B	109.5
N1—Cu1—S3^i^	119.76 (9)	N5—C13—H13C	109.5
S2—Cu1—S3^i^	93.84 (3)	H13A—C13—H13C	109.5
N1—Cu1—S1	85.99 (9)	H13B—C13—H13C	109.5
S2—Cu1—S1	113.14 (3)	N5—C14—C15	102.7 (3)
S3^i^—Cu1—S1	112.85 (3)	N5—C14—H14A	111.2
N4—Cu2—S3	134.56 (8)	C15—C14—H14A	111.2
N4—Cu2—S1	117.78 (8)	N5—C14—H14B	111.2
S3—Cu2—S1	92.23 (3)	C15—C14—H14B	111.2
N4—Cu2—S2	86.45 (8)	H14A—C14—H14B	109.1
S3—Cu2—S2	113.28 (3)	N6—C15—C14	102.9 (3)
S1—Cu2—S2	113.61 (3)	N6—C15—H15A	111.2
N7—Cu3—S1^i^	134.18 (9)	C14—C15—H15A	111.2
N7—Cu3—S2	119.29 (9)	N6—C15—H15B	111.2
S1^i^—Cu3—S2	91.71 (3)	C14—C15—H15B	111.2
N7—Cu3—S3	86.41 (9)	H15A—C15—H15B	109.1
S1^i^—Cu3—S3	113.47 (3)	N6—C16—H16A	109.5
S2—Cu3—S3	112.56 (3)	N6—C16—H16B	109.5
C11—S1—Cu3^i^	116.61 (11)	H16A—C16—H16B	109.5
C11—S1—Cu2	112.10 (11)	N6—C16—H16C	109.5
Cu3^i^—S1—Cu2	110.12 (4)	H16A—C16—H16C	109.5
C11—S1—Cu1	92.50 (12)	H16B—C16—H16C	109.5
Cu3^i^—S1—Cu1	65.70 (3)	N4—C17—C18	123.0 (3)
Cu2—S1—Cu1	65.26 (3)	N4—C17—C22	119.5 (3)
C22—S2—Cu1	115.66 (11)	C18—C17—C22	117.4 (3)
C22—S2—Cu3	115.33 (11)	C19—C18—C17	121.7 (3)
Cu1—S2—Cu3	109.54 (4)	C19—C18—H18A	119.2
C22—S2—Cu2	93.03 (12)	C17—C18—H18A	119.2
Cu1—S2—Cu2	65.89 (3)	C18—C19—C20	120.3 (4)
Cu3—S2—Cu2	66.17 (3)	C18—C19—H19A	119.9
C33—S3—Cu1^i^	113.41 (11)	C20—C19—H19A	119.9
C33—S3—Cu2	118.10 (12)	C21—C20—C19	119.4 (4)
Cu1^i^—S3—Cu2	108.65 (3)	C21—C20—H20A	120.3
C33—S3—Cu3	92.50 (12)	C19—C20—H20A	120.3
Cu1^i^—S3—Cu3	65.86 (3)	C20—C21—C22	121.1 (3)
Cu2—S3—Cu3	66.04 (3)	C20—C21—H21A	119.5
C1—N1—C6	120.3 (3)	C22—C21—H21A	119.5
C1—N1—Cu1	117.8 (2)	C21—C22—C17	120.2 (3)
C6—N1—Cu1	117.9 (2)	C21—C22—S2	117.8 (3)
C1—N2—C2	124.9 (3)	C17—C22—S2	122.0 (3)
C1—N2—C3	111.6 (3)	N7—C23—N8	122.1 (3)
C2—N2—C3	121.4 (3)	N7—C23—N9	127.5 (3)
C1—N3—C5	124.3 (3)	N8—C23—N9	110.4 (3)
C1—N3—C4	110.7 (3)	N8—C24—H24A	109.5
C5—N3—C4	119.4 (3)	N8—C24—H24B	109.5
C12—N4—C17	120.5 (3)	H24A—C24—H24B	109.5
C12—N4—Cu2	118.8 (2)	N8—C24—H24C	109.5
C17—N4—Cu2	117.5 (2)	H24A—C24—H24C	109.5
C12—N5—C13	126.2 (3)	H24B—C24—H24C	109.5
C12—N5—C14	111.9 (3)	N8—C25—C26	102.7 (3)
C13—N5—C14	121.7 (3)	N8—C25—H25A	111.2
C12—N6—C16	126.7 (3)	C26—C25—H25A	111.2
C12—N6—C15	111.3 (3)	N8—C25—H25B	111.2
C16—N6—C15	121.0 (3)	C26—C25—H25B	111.2
C23—N7—C28	120.0 (3)	H25A—C25—H25B	109.1
C23—N7—Cu3	117.7 (2)	N9—C26—C25	102.3 (3)
C28—N7—Cu3	117.1 (2)	N9—C26—H26A	111.3
C23—N8—C24	123.9 (3)	C25—C26—H26A	111.3
C23—N8—C25	110.6 (3)	N9—C26—H26B	111.3
C24—N8—C25	120.5 (3)	C25—C26—H26B	111.3
C23—N9—C27	122.2 (3)	H26A—C26—H26B	109.2
C23—N9—C26	109.0 (3)	N9—C27—H27A	109.5
C27—N9—C26	116.2 (3)	N9—C27—H27B	109.5
N1—C1—N2	123.1 (3)	H27A—C27—H27B	109.5
N1—C1—N3	126.5 (3)	N9—C27—H27C	109.5
N2—C1—N3	110.3 (3)	H27A—C27—H27C	109.5
N2—C2—H2A	109.5	H27B—C27—H27C	109.5
N2—C2—H2B	109.5	N7—C28—C33	120.4 (3)
H2A—C2—H2B	109.5	N7—C28—C29	122.7 (3)
N2—C2—H2C	109.5	C33—C28—C29	116.7 (3)
H2A—C2—H2C	109.5	C30—C29—C28	121.8 (4)
H2B—C2—H2C	109.5	C30—C29—H29A	119.1
N2—C3—C4	103.3 (3)	C28—C29—H29A	119.1
N2—C3—H3A	111.1	C29—C30—C31	120.8 (3)
C4—C3—H3A	111.1	C29—C30—H30A	119.6
N2—C3—H3B	111.1	C31—C30—H30A	119.6
C4—C3—H3B	111.1	C30—C31—C32	119.7 (4)
H3A—C3—H3B	109.1	C30—C31—H31A	120.1
N3—C4—C3	103.9 (3)	C32—C31—H31A	120.1
N3—C4—H4A	111.0	C31—C32—C33	120.4 (3)
C3—C4—H4A	111.0	C31—C32—H32A	119.8
N3—C4—H4B	111.0	C33—C32—H32A	119.8
C3—C4—H4B	111.0	C32—C33—C28	120.6 (3)
H4A—C4—H4B	109.0	C32—C33—S3	117.7 (3)
N3—C5—H5A	109.5	C28—C33—S3	121.7 (3)
N3—C5—H5B	109.5	F2—P1—F3	92.8 (3)
H5A—C5—H5B	109.5	F2—P1—F4	90.9 (2)
N3—C5—H5C	109.5	F3—P1—F4	93.1 (2)
H5A—C5—H5C	109.5	F2—P1—F1	176.4 (3)
H5B—C5—H5C	109.5	F3—P1—F1	90.5 (3)
N1—C6—C11	120.4 (3)	F4—P1—F1	90.6 (2)
N1—C6—C7	122.7 (3)	F2—P1—F6	90.8 (2)
C11—C6—C7	116.7 (3)	F3—P1—F6	89.5 (2)
C8—C7—C6	121.5 (4)	F4—P1—F6	176.8 (2)
C8—C7—H7A	119.3	F1—P1—F6	87.5 (2)
C6—C7—H7A	119.3	F2—P1—F5	88.3 (2)
C7—C8—C9	120.9 (3)	F3—P1—F5	178.7 (2)
C7—C8—H8A	119.6	F4—P1—F5	87.59 (19)
C9—C8—H8A	119.6	F1—P1—F5	88.4 (2)
C8—C9—C10	119.3 (4)	F6—P1—F5	89.74 (19)
C8—C9—H9A	120.3	Cl1—C100—Cl2	116.3 (3)
C10—C9—H9A	120.3	Cl1—C100—H10B	108.2
C9—C10—C11	120.2 (3)	Cl2—C100—H10B	108.2
C9—C10—H10A	119.9	Cl1—C100—H10C	108.2
C11—C10—H10A	119.9	Cl2—C100—H10C	108.2
C10—C11—C6	121.3 (3)	H10B—C100—H10C	107.4
C10—C11—S1	117.0 (3)	N100—C101—C102	176.3 (7)
C6—C11—S1	121.6 (3)	C101—C102—H10D	109.5
N4—C12—N6	128.3 (3)	C101—C102—H10E	109.5
N4—C12—N5	122.1 (3)	H10D—C102—H10E	109.5
N6—C12—N5	109.6 (3)	C101—C102—H10F	109.5
N5—C13—H13A	109.5	H10D—C102—H10F	109.5
N5—C13—H13B	109.5	H10E—C102—H10F	109.5
			
N4—Cu2—S1—C11	−5.52 (16)	C1—N1—C6—C11	−146.9 (3)
S3—Cu2—S1—C11	−150.02 (13)	Cu1—N1—C6—C11	10.2 (4)
S2—Cu2—S1—C11	93.33 (13)	C1—N1—C6—C7	36.9 (5)
N4—Cu2—S1—Cu3^i^	−137.09 (9)	Cu1—N1—C6—C7	−166.0 (3)
S3—Cu2—S1—Cu3^i^	78.41 (4)	N1—C6—C7—C8	178.4 (3)
S2—Cu2—S1—Cu3^i^	−38.24 (5)	C11—C6—C7—C8	2.1 (5)
N4—Cu2—S1—Cu1	−87.73 (9)	C6—C7—C8—C9	−0.1 (6)
S3—Cu2—S1—Cu1	127.77 (3)	C7—C8—C9—C10	−0.9 (6)
S2—Cu2—S1—Cu1	11.11 (3)	C8—C9—C10—C11	−0.2 (5)
N1—Cu1—S1—C11	9.32 (13)	C9—C10—C11—C6	2.4 (5)
S2—Cu1—S1—C11	−124.97 (11)	C9—C10—C11—S1	−177.8 (3)
S3^i^—Cu1—S1—C11	130.02 (11)	N1—C6—C11—C10	−179.7 (3)
N1—Cu1—S1—Cu3^i^	−108.86 (9)	C7—C6—C11—C10	−3.3 (5)
S2—Cu1—S1—Cu3^i^	116.85 (3)	N1—C6—C11—S1	0.5 (4)
S3^i^—Cu1—S1—Cu3^i^	11.84 (3)	C7—C6—C11—S1	176.9 (3)
N1—Cu1—S1—Cu2	122.56 (9)	Cu3^i^—S1—C11—C10	−123.8 (2)
S2—Cu1—S1—Cu2	−11.73 (3)	Cu2—S1—C11—C10	108.0 (3)
S3^i^—Cu1—S1—Cu2	−116.74 (3)	Cu1—S1—C11—C10	172.2 (2)
N1—Cu1—S2—C22	−13.93 (18)	Cu3^i^—S1—C11—C6	56.0 (3)
S3^i^—Cu1—S2—C22	−150.72 (13)	Cu2—S1—C11—C6	−72.2 (3)
S1—Cu1—S2—C22	92.41 (13)	Cu1—S1—C11—C6	−7.9 (3)
N1—Cu1—S2—Cu3	−146.33 (12)	C17—N4—C12—N6	−36.2 (5)
S3^i^—Cu1—S2—Cu3	76.88 (4)	Cu2—N4—C12—N6	123.1 (3)
S1—Cu1—S2—Cu3	−39.98 (5)	C17—N4—C12—N5	146.0 (3)
N1—Cu1—S2—Cu2	−95.26 (12)	Cu2—N4—C12—N5	−54.7 (4)
S3^i^—Cu1—S2—Cu2	127.95 (3)	C16—N6—C12—N4	−11.8 (6)
S1—Cu1—S2—Cu2	11.08 (3)	C15—N6—C12—N4	179.4 (3)
N7—Cu3—S2—C22	6.66 (16)	C16—N6—C12—N5	166.2 (3)
S1^i^—Cu3—S2—C22	151.36 (13)	C15—N6—C12—N5	−2.5 (4)
S3—Cu3—S2—C22	−92.29 (13)	C13—N5—C12—N4	−2.4 (6)
N7—Cu3—S2—Cu1	139.22 (10)	C14—N5—C12—N4	172.1 (3)
S1^i^—Cu3—S2—Cu1	−76.07 (4)	C13—N5—C12—N6	179.4 (3)
S3—Cu3—S2—Cu1	40.28 (4)	C14—N5—C12—N6	−6.1 (4)
N7—Cu3—S2—Cu2	88.31 (10)	C12—N5—C14—C15	11.4 (4)
S1^i^—Cu3—S2—Cu2	−126.98 (3)	C13—N5—C14—C15	−173.8 (3)
S3—Cu3—S2—Cu2	−10.63 (3)	C12—N6—C15—C14	9.3 (4)
N4—Cu2—S2—C22	−9.82 (13)	C16—N6—C15—C14	−160.2 (3)
S3—Cu2—S2—C22	127.79 (11)	N5—C14—C15—N6	−11.8 (4)
S1—Cu2—S2—C22	−128.67 (11)	C12—N4—C17—C18	−31.7 (5)
N4—Cu2—S2—Cu1	107.01 (8)	Cu2—N4—C17—C18	168.8 (3)
S3—Cu2—S2—Cu1	−115.38 (3)	C12—N4—C17—C22	153.1 (3)
S1—Cu2—S2—Cu1	−11.83 (3)	Cu2—N4—C17—C22	−6.5 (4)
N4—Cu2—S2—Cu3	−126.24 (8)	N4—C17—C18—C19	−175.3 (3)
S3—Cu2—S2—Cu3	11.36 (3)	C22—C17—C18—C19	0.0 (5)
S1—Cu2—S2—Cu3	114.91 (3)	C17—C18—C19—C20	0.3 (6)
N4—Cu2—S3—C33	19.03 (17)	C18—C19—C20—C21	0.0 (6)
S1—Cu2—S3—C33	152.89 (13)	C19—C20—C21—C22	−0.7 (6)
S2—Cu2—S3—C33	−90.17 (13)	C20—C21—C22—C17	1.0 (6)
N4—Cu2—S3—Cu1^i^	149.98 (11)	C20—C21—C22—S2	−179.4 (3)
S1—Cu2—S3—Cu1^i^	−76.16 (4)	N4—C17—C22—C21	174.8 (3)
S2—Cu2—S3—Cu1^i^	40.78 (4)	C18—C17—C22—C21	−0.7 (5)
N4—Cu2—S3—Cu3	98.47 (11)	N4—C17—C22—S2	−4.8 (4)
S1—Cu2—S3—Cu3	−127.67 (3)	C18—C17—C22—S2	179.7 (3)
S2—Cu2—S3—Cu3	−10.73 (3)	Cu1—S2—C22—C21	126.2 (3)
N7—Cu3—S3—C33	10.70 (14)	Cu3—S2—C22—C21	−104.1 (3)
S1^i^—Cu3—S3—C33	−126.53 (11)	Cu2—S2—C22—C21	−169.1 (3)
S2—Cu3—S3—C33	131.02 (11)	Cu1—S2—C22—C17	−54.1 (3)
N7—Cu3—S3—Cu1^i^	125.29 (9)	Cu3—S2—C22—C17	75.5 (3)
S1^i^—Cu3—S3—Cu1^i^	−11.94 (3)	Cu2—S2—C22—C17	10.5 (3)
S2—Cu3—S3—Cu1^i^	−114.39 (3)	C28—N7—C23—N8	−153.4 (3)
N7—Cu3—S3—Cu2	−109.07 (9)	Cu3—N7—C23—N8	52.6 (4)
S1^i^—Cu3—S3—Cu2	113.70 (3)	C28—N7—C23—N9	28.1 (5)
S2—Cu3—S3—Cu2	11.25 (3)	Cu3—N7—C23—N9	−125.9 (3)
S2—Cu1—N1—C1	−96.4 (3)	C24—N8—C23—N7	20.5 (5)
S3^i^—Cu1—N1—C1	31.7 (3)	C25—N8—C23—N7	175.5 (3)
S1—Cu1—N1—C1	145.8 (3)	C24—N8—C23—N9	−160.8 (3)
S2—Cu1—N1—C6	105.9 (2)	C25—N8—C23—N9	−5.8 (4)
S3^i^—Cu1—N1—C6	−126.0 (2)	C27—N9—C23—N7	29.0 (6)
S1—Cu1—N1—C6	−11.9 (2)	C26—N9—C23—N7	169.2 (3)
S3—Cu2—N4—C12	90.9 (3)	C27—N9—C23—N8	−149.6 (3)
S1—Cu2—N4—C12	−34.6 (3)	C26—N9—C23—N8	−9.4 (4)
S2—Cu2—N4—C12	−149.5 (2)	C23—N8—C25—C26	17.7 (4)
S3—Cu2—N4—C17	−109.2 (2)	C24—N8—C25—C26	173.6 (4)
S1—Cu2—N4—C17	125.3 (2)	C23—N9—C26—C25	19.5 (4)
S2—Cu2—N4—C17	10.4 (2)	C27—N9—C26—C25	162.4 (3)
S1^i^—Cu3—N7—C23	−97.8 (3)	N8—C25—C26—N9	−21.5 (4)
S2—Cu3—N7—C23	28.6 (3)	C23—N7—C28—C33	−145.3 (3)
S3—Cu3—N7—C23	142.5 (3)	Cu3—N7—C28—C33	8.9 (4)
S1^i^—Cu3—N7—C28	107.5 (2)	C23—N7—C28—C29	40.3 (5)
S2—Cu3—N7—C28	−126.2 (2)	Cu3—N7—C28—C29	−165.5 (3)
S3—Cu3—N7—C28	−12.2 (2)	N7—C28—C29—C30	175.1 (3)
C6—N1—C1—N2	−152.7 (3)	C33—C28—C29—C30	0.6 (5)
Cu1—N1—C1—N2	50.2 (4)	C28—C29—C30—C31	0.0 (6)
C6—N1—C1—N3	31.1 (5)	C29—C30—C31—C32	−0.3 (6)
Cu1—N1—C1—N3	−126.0 (3)	C30—C31—C32—C33	−0.1 (6)
C2—N2—C1—N1	16.7 (6)	C31—C32—C33—C28	0.7 (5)
C3—N2—C1—N1	−179.7 (3)	C31—C32—C33—S3	−178.3 (3)
C2—N2—C1—N3	−166.5 (4)	N7—C28—C33—C32	−175.6 (3)
C3—N2—C1—N3	−2.9 (4)	C29—C28—C33—C32	−0.9 (5)
C5—N3—C1—N1	23.3 (6)	N7—C28—C33—S3	3.3 (4)
C4—N3—C1—N1	176.4 (3)	C29—C28—C33—S3	178.0 (3)
C5—N3—C1—N2	−153.3 (4)	Cu1^i^—S3—C33—C32	103.8 (3)
C4—N3—C1—N2	−0.2 (4)	Cu2—S3—C33—C32	−127.5 (2)
C1—N2—C3—C4	4.6 (4)	Cu3—S3—C33—C32	168.5 (3)
C2—N2—C3—C4	168.8 (4)	Cu1^i^—S3—C33—C28	−75.2 (3)
C1—N3—C4—C3	3.0 (4)	Cu2—S3—C33—C28	53.6 (3)
C5—N3—C4—C3	157.6 (3)	Cu3—S3—C33—C28	−10.5 (3)
N2—C3—C4—N3	−4.4 (4)		

Symmetry code: (i) −*x*+1, −*y*+1, −*z*.

### Hydrogen-bond geometry (Å, º)

**Table:**

*D*—H···*A*	*D*—H	H···*A*	*D*···*A*	*D*—H···*A*
C31—H31*A*···F6^i^	0.95	2.49	3.290 (5)	141
C102—H10*F*···F4^ii^	0.98	2.43	3.173 (6)	133
C102—H10*E*···F1^iii^	0.98	2.44	3.187 (7)	133

Symmetry codes: (i) −*x*+1, −*y*+1, −*z*; (ii) −*x*+1, −*y*+1, −*z*+1; (iii) *x*−1, *y*+1, *z*.
